# Comparative study between clinical history and polysomnogram in the obstructive sleep apnea / hypopnea syndrome

**DOI:** 10.1016/S1808-8694(15)31168-X

**Published:** 2015-10-19

**Authors:** Lys Maria Allenstein Gondim, Luciana Matshie Matumoto, Marco Antônio Cezário de Melo J únior, Sérgio Bittencourt, Ulisses José Ribeiro

**Affiliations:** 1 MD. Otorhinolaryngologist, Former Resident of the Otorhinolaryngology Service - Hospital Nossa Senhora de Lourdes - SP; Professor and ENT - Universidade do Vale do Itajaí, UNIVALI. & Hospital Universitário Infantil Pequeno Anjo, HUPA; 2 MD. Former Resident of the Otorhinolaryngology Service - Hospital Nossa Senhora de Lourdes - SP; 3 MD. Resident of the Otorhinolaryngology Service - Hospital Nossa Senhora de Lourdes - SP; 4 MD. Otorhinolaryngologist, Head of the Otorhinolaryngology Service - Hospital Nossa Senhora de Lourdes - SP; 5 MD. Otorhinolaryngologist, Head of the Otorhinolaryngology Service - Hospital Nossa Senhora de Lourdes - SP; Hospital Nossa Senhora de Lourdes, São Paulo SP

**Keywords:** apnea, epworth, polysomnogram, snoring

## Abstract

Recognizing sleep-disordered breathing is on the rise every year. Manifestations, such as snoring, that were earlier considered mere inconvenients are now acquiring greater importance concerning life quality and social impact. **Aim** of the study: To compare the clinical history to polysomnogram (PSG) results in the Obstructive Sleep Apnea/Hypopnea Syndrome (OSAHS).

**Materials and Methods:**

125 patients were analyzed, in a retrospective study. Specific questionnaires, avaliations of Body Mass Index and Epworth Scale were carried out.

**Results:**

Among the patients, 75 were males and 50 were females. The main symptom was snoring. 46% had normal PSG, 30% had light OSAHS, 15% moderate and 9% severe OSAHS and it was not observed a correlation between clinical data and PSG results. Concerning clinical symptoms, only insomnia has shown relevance when univariably analyzed in normal and light OSAHS patients (p<0,05) compared to patients with moderate and severe OSAHS, losing its importance when analyzed together with other factors.

**Conclusion:**

the clinical history, per se, is not sufficient to define OSAHS' diagnosis or it's severity.

## INTRODUCTION

Obstructive Sleep Apnea is a condition characterized by a repetitive upper airway obstruction for 10 seconds or more, frequently resulting in oxygen desaturation and sleep disorders. The classical manifestation is daily sleepiness, however, other symptoms such as snoring, restless sleep, low concentration and fatigue are commonly reported^1^.

Clinical evaluation must involve the patient and his family^2^, focusing the story on the main complaint and in quantitative questionnaires, by means of established sleepiness scales, such as the Epworth^3^, ^4^, ^5^, ^6^, ^7^.

Physical exam is also important to predict the sleep obstructive apnea syndrome (SOAS)^8^. Body Mass Index (BMI) is but one of the data to be analyzed. Numerous alterations have been pinned to sleep disorders, such as cardiovascular, which have a close relationship with SAOS, especially pulmonary hypertension and high blood pressure, and cases of acute myocardial infarction^9^,^10^, and such facts only come to stress the need for more detailed studies about this disease.

As to complementary diagnostic tests, Polysomnography (PSG) is considered the gold standard used to define sleep disorders^2^. During the procedure, the following is recorded: sleep stage and continuity, respiratory effort, oxygen saturation, body position, EKG and body movements. Other tests such as cephalometry, nasal endoscopy with Müller's maneuver, video-fluoroscopy, computerized tomography and MRI may also help in diagnosis.

The major goal of this paper is to compare data from the clinical history with polysomnography results, in cases of OSAS suspicion.

## MATERIALS AND METHODS

We ran a historical, transversal, cohort study with 125 patients who were submitted to polysomnography in the Department of Otorhinolaryngology of the Nossa Senhora de Lourdes Hospital, from November of 2003 to February of 2004, ordered due to suspicion of OSAS. This research project was approved by the Ethics Committee of the Hospital. After signed informed consents, a questionnaire was given to the patients, assessing Epworth's scale (Table 1) and the major complaints were: snoring, apnea, daily sleepiness, insomnia, dyspepsia, anxiety, attention and concentration deficits, restless sleep, parasomnia and early morning headache. We also assessed previous diseases the patient had.

**Table 1:** Epworth's Scale

	No chance of napping (0)	Light chance of napping (1)	Moderate chance of napping (2)	High chance of napping (3)
I. Seated and Reading				
II. Watching TV				
III. Seated in a public place				
IV. As a passenger in a car, bus or train, in one hour				
V. Laying down to rest in the afternoon, when possible				
VI. Seated and talking to someone				
VII. Seated calmly after lunch, without drinking alcohol				
VIII. In the car, in intense traffic, when the car stops				

Other data from identification and physical exams were compared (gender, age, weight and height ratio - BMI). The data were then correlated with the results from polysomnography. The patients were submitted to polysomnography exam under the device Sonolab 620®, with 8 EKG channels, with biological signs recording (eye movements; leg movements; chest and abdominal bands and nasal airflow in order to monitor the entire respiration; microphone to detect snoring level; abdominal movement and nasal air flow for complete breathing monitoring; microphone to detect snoring, electroencephalogram; electromyogram; oximetry and body position).

For statistical analysis purposes, for each one of the symptoms, we initially tested the null hypothesis - the number of cases with polysomnographic result of moderate to severe OSAS among those with the symptom is equal to the number of cases with results of OSAS results of moderate to severe intensity, among those who do not have the symptom, versus the alternative hypothesis of different ratios. For that we used Fisher's Exact Test. Following that, we carried out a joint analysis of all the symptoms. In such a case, we tested the same hypothesis aforesaid, but in this approach we took the other variables into account. Therefore we adjusted a Logistics Regression model, considering Wald's test to assess the hypotheses. In all the tests a p value of p<0,05 was considered as statistically significant. For this analysis the patients in the study were broken down into two groups: the first with individuals deemed normal and with mild OSAS, and the second with patients who had moderate and severe OSAS seen at the polysomnography.

## RESULTS

75 (60%) of the patients in the study were males and 50 (40%) were females. They were distributed in regards to their age in 6 groups: below 15 years (6 patients), 15 to 24 years old (2 patients), 25 to 34 years old (16 patients) 35 to 44 years (37 patients) 45 to 54 years (34 patients) and above 55 years (30 patients).

As to the results from polysomnography, 57 patients (46%) had apnea/hypopnea ratio (AHR) within normal values (< 5 per hour - snorers), 38 patients (30%) had mild OSAS (AHR between 5 and 15 per hour), 19 patients (15%) had moderate OSAS (AHR between 15 and 30 per hour) and 11 patients (9%) with severe OSAS (AHR > 30 per hour) (Table 2).

**Table 2:** Distribution as to OSAS severity

AHI	N=125	%
0 – 5	57	46%
5 – 15	38	30%
15 – 30	19	15%
> 30	11	9%
TOTAL	125	100%

AHI: Apnea and hypopnea index per hour of sleep

N: number of patients

**Graph 1:**
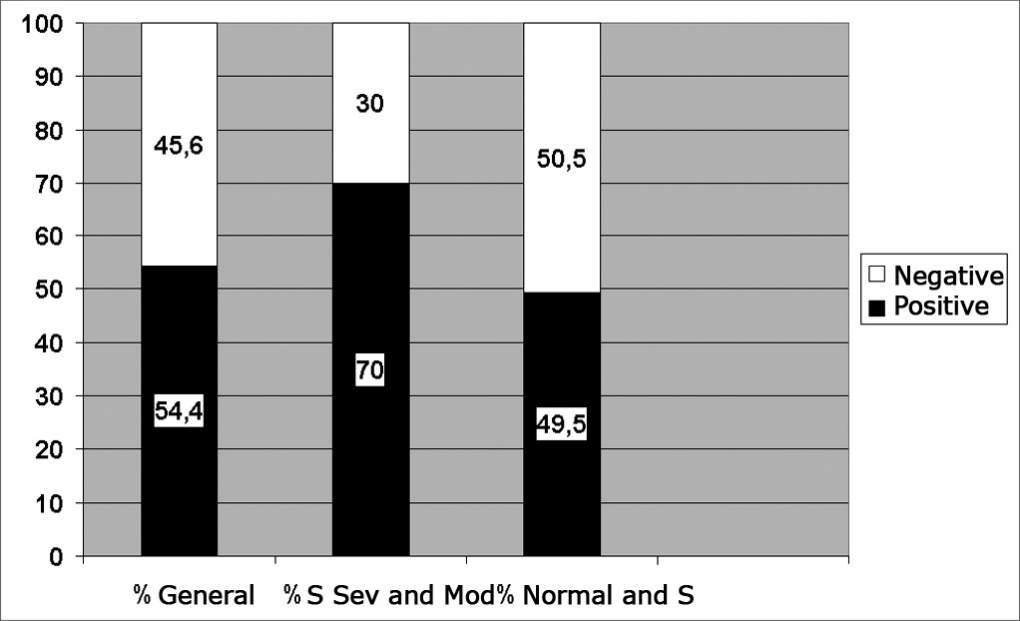
Epworth's Index percentage distribution according to results from polysomnographies

We compared the results from the Epworth's scale (Table 2) divided in positive for values above 10, according to Johns^1^,^5^, ^6^, ^7^; with the polysomnographic findings divided in normal patients and those with mild OSAS, compared to those patients with moderate and severe OSAS, where, among them the scale was positive in 70% (Graph 1).

As to the relation between BMI and AHR: in the study with the 125 patients, 17 had BMI above 36, three had AHR below 5 (normal), making up only 5.3% of the 57 patients evaluated considered normal. Seven out of the 17 patients had mild OSAS according to the polysomnography, corresponding to 18.4% of the 38 patients with mild OSAS in the study. Two patients had moderate OSAS, representing 10.5% of the 19 patients with moderate OSAS and 5 patients of the 17 with BMI above 36 had severe

S: OSAS Sev.: severe Mod.: moderate

**Graph 2:**
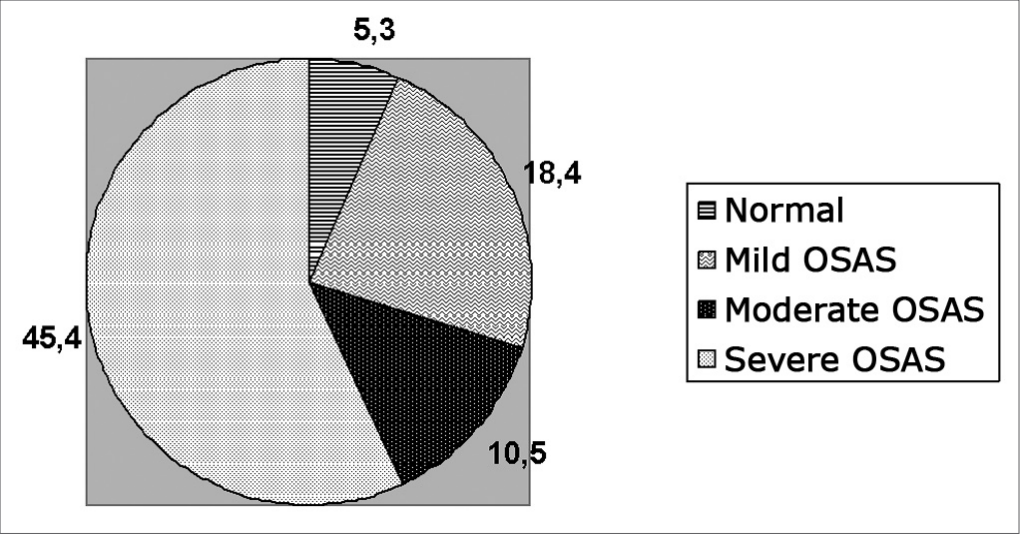
Percentage of patients with BMI > 36 in relation to results from polysomnographies

OSAS, corresponding to 45.4% of the 11 patients with severe OSAS in the study (Graph 2).

The major clinical complaints the patients had, according to the interview were broken down in two groups in regards of the PSG results: patients with AHR below 15 (normal or with mild OSAS) and with AHR above 15 (moderate or severe OSAS). Most patients complained of snoring, apnea, excessive daily sleepiness (EDS), insomnia, dyspepsia, stress, lack of concentration and attention, restless sleep, parasomnia, and morning headache, broken down according to Table 3.

**Table 3:** Association between the patients' clinical complaints and the polysomnographic results

	N	% total	Moderate and severe OSAS	Moderate and severe OSAS	N Mild and Normal OSAS	% Mild and Normal OSAS	p
Snoring	104	83.2	26	86.7	78	82.1	0.7802
Apnea	45	36	11	36.7	34	35.8	1
EDS	66	52.8	18	60	48	50.5	0.4068
Insomnia	36	28.8	03	10	33	34.7	0.0102
Dyspepsia	53	42.4	15	50	38	40	0.3984
Stress	75	60	15	50	60	63.2	0.2084
Lack of concentration	49	36.2	10	33.3	39	41	0.5235
Restless sleep	61	48.8	10	33.3	51	53.7	0.0612
Parasomnia	27	21.6	06	20	21	22.1	1
Morning headache	18	14.4	04	13.3	14	14.7	1
Other	06	4.8	03	10	03	3.2	0.1489
TOTAL number of patients	125		30		95		

N: number of patients

EDS: excess daily sleepiness

According to the statistical analysis of results regarding the correlation between these complaints and the clinical findings with the results from polysomnography were the following: in Univaried Analysis (Fisher's Exact Test): high values for snoring (p=0.78), apnea (p=1), ESD (p=0.40), insomnia (p=0.01), dyspepsia (p=0.39), stress (p=0.20), lack of concentration (p=0.52), restless sleep (p=0.06), parasomnia (p=1), morning headaches (p=1), others (p=0.14), BMI (p=0.26) and Epworth's scale (p=0.06). In multivaried analysis (hypotheses tested for each one of the signs and symptoms in the presence of the others): snoring (p=0.87), apnea (p=0.70), ESD (p=0.99), insomnia (p=0.07), dyspepsia (p=0,25), stress (p=0.27) lack of concentration and attention (p=0.87), restless sleep (p=0.10), parasomnia (p=0.95), morning headaches (p=0.90), others (p=0.70), BMI (p=0.27) and Epworth's scale (p=0.27).

## DISCUSSION

Clinical history and the physical exam are fundamental in OSAS diagnosis^1^,^2^,^7^,^8^. Snoring, restless sleep, contraction reduction, insomnia, among other findings in the clinical history, including using the Epworth's scale are also considered important in the anamneses of patients with sleep respiratory disorders. As far as physical signs are concerned, a high BMI (indicating overweight/obesity), tonsil hypertrophy, hypertrophy of palate and tongue are also considered relevant^8^.

However, according to Pastor et al., clinical history did not prove enough for a full diagnosis of sleep disorders^11^. According to this author, associated diseases present reduce the value of many screening complementary methods, thus it is fundamental to have the patient undergo polysomnography in order to fully assess the problem, not thinking about OSAS only.

In the present investigation, clinical complaint, BMI and the Epworth's scale were correlated with the results from the polysomnography studies. We noticed that there were no statistically significant differences (p>0.05) among the symptoms related to daily sleepiness, apnea, lack of concentration and attention, restless sleep, as well as findings of high BMI and positive Epworth scales, all comparative data with the results from polysomnographies classified as moderate to severe, versus the ones considered normal or mild. Complaints such as lack of concentration and restless sleep were more prevalent in normal patients and those with mild OSAS, although they did not show statistical meaning (p>0.05). Another

**Graph 3:**
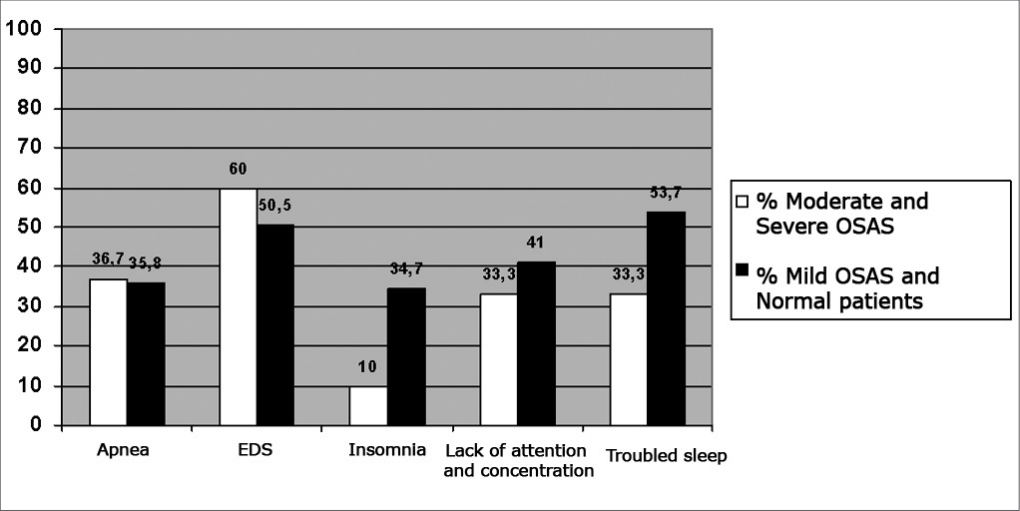
Percentage relationship of clinical complaints among patients with Severe and Moderate OSAS X Mild OSAS and patients with normal polysomnographies

important finding were insomnia, described as an unusual symptom in patients with OSAS^1^,^2^. In our statistics, it was also significant, however, in normal patients and in those with mild OSAS (p<0.05) and not in those with moderate and severe OSAS, as advocated in the literature, and only when analyzed alone, losing its importance when in the presence of other evaluated signs and symptoms (Graph 3).

## CONCLUSION

The present investigation showed that clinical history and physical exam should not be considered alone when one is dealing with obstructive sleep respiratory disorders, that means that the physician should not consider only these findings in order to try and define the presence and/or degree of severity of the cases suspected of being OSAS.

## References

[1] Flemons WW, Buysse D (1999). The Report of American Academy of Sleep Medicine Task Force.. Sleep Related Breathing Disorders in Adults: Recommendations for Syndrome Definition and Measurement Techniques in Clinical Research. Sleep.

[2] Fujita RR, Moysés MG, Vuono IM (2003). Ronco e Apnéia do Sono.. In: Tratado de Otorrinolaringologia da Sociedade Brasileira de Otorrinolaringologia.

[3] Keenan S.A, S Chokroverty (1999). Sleep disorders medicine: basic science, technical considerations and clinical aspects..

[4] Mitler MM, Carskadon MA, Hirshkowitz M, MH Kryger, T Roth, WC Dement (2000). Principles and practice of sleep medicine..

[5] Johns MW (1991). A new method for measuring daytime sleepiness: the Epworth Sleepiness Scale.. Sleep.

[6] Johns MW (1992). Reliability and factor analysis of the Epworth Sleepiness Scale.. Sleep.

[7] Oleejniczak PW, Fisch BJ (2003). Sleep disorders.. Med Clin North Am.

[8] Zonato AI, Bittencourt LR, Martinho FL, Júnior JF, Gregório LC, Tufik KS (2003). Association of systemic head and neck physical examination with severity of obstructive sleep apnea-hypopnea syndrome.. Laryngoscope.

[9] Cavallari FEM, Leite MGJ, Mestriner PRE, Couto LGF, Fomin DS, Oliveira JAA (2002). Relação entre hipertensão arterial sistêmica e síndrome da apnéia obstrutiva do sono.. In: Rev Bras Otorrinolaringol.

[10] Dart RA, Gregoire JR, Gutterman DD, Woolf SH (2003). The Association of Hipertension and Secondary Cardiovascular Disease With Sleep-Disorder Breathing.. Chest.

[11] Pastor J, Fernández-Lorente J, Ortega B, Galán JM (2001). Análisis comparativo de la historia clínica y la polisomnografia en la patologia del sueño.. Relevancia diagnóstica de la polisomnografia. Rev Neurol Jan.

